# Prevalence of Gestational Diabetes Mellitus and Its Risk Factors in Chinese Pregnant Women: A Prospective Population-Based Study in Tianjin, China

**DOI:** 10.1371/journal.pone.0121029

**Published:** 2015-03-23

**Authors:** Junhong Leng, Ping Shao, Cuiping Zhang, Huiguang Tian, Fuxia Zhang, Shuang Zhang, Ling Dong, Lili Li, Zhijie Yu, Juliana C. N. Chan, Gang Hu, Xilin Yang

**Affiliations:** 1 Department of Epidemiology and Biostatistics, School of Public Health, Tianjin Medical University, Tianjin, China; 2 Tianjin Women and Children’s Health Centre, Tianjin, China; 3 Population Cancer Research Program and Department of Pediatrics, Dalhousie University, Halifax, Canada; 4 Department of Medicine and Therapeutics, Hong Kong Institute of Diabetes and Obesity, International Diabetes Federation Centre of Education, The Chinese University of Hong Kong-Prince of Wales Hospital-International Diabetes Federation Centre of Education, Hong Kong SAR, China; 5 Chronic Disease Epidemiology Laboratory, Pennington Biomedical Research Center, Baton Rouge, Louisiana, United States of America; University of Tolima, COLOMBIA

## Abstract

**Objective:**

We compared the increases in the prevalence of gestational diabetes mellitus (GDM) based on the 1999 World Health Organization (WHO) criteria and its risk factors in Tianjin, China, over a 12-year period. We also examined the changes in the prevalence using the criteria of International Association of Diabetes and Pregnancy Study Group (IADPSG).

**Methods:**

In 2010-2012, 18589 women who registered within 12 weeks of gestation underwent a glucose challenge test (GCT) at 24-28 gestational weeks. Amongst them, 2953 women with 1-hour plasma glucose ≥7.8 mmol/L underwent a 75-gram 2-hour oral glucose tolerance test (OGTT) and 781 women had a positive GCT but absented from the standard OGTT. An adjusted prevalence of GDM was calculated for the whole cohort of women by including an estimate of the proportion of women with positive GCTs who did not have OGTTs but would have been expected to have GDM. Logistic regression was used to obtain odds ratios and 95% confidence intervals using the IADPSG criteria. The prevalence of GDM risk factors was compared to the 1999 survey.

**Results:**

The adjusted prevalence of GDM by the 1999 WHO criteria was 8.1%, a 3.5-fold increase as in 1999. Using the IADPSG criteria increased the adjusted prevalence further to 9.3%. Advanced age, higher pre-pregnancy body mass index, Han-nationality, higher systolic blood pressure (BP), a family history of diabetes, weight gain during pregnancy and habitual smoking were risk factors for GDM. Compared to the 1999 survey, the prevalence of overweight plus obesity had increased by 1.8 folds, age≥30 years by 2.3 folds, systolic BP by 2.3 mmHg over the 12-year period.

**Conclusions:**

Increasing prevalence of overweight/obesity and older age at pregnancy were accompanied by increasing prevalence of GDM, further increased by change in diagnostic criteria.

## Introduction

Gestational diabetes mellitus (GDM) is a common condition during pregnancy which is associated with negative short-term and long-term outcomes both for mothers and their offspring. Apart from having increased risk of preeclampsia during pregnancy, women with GDM had high risks for type 2 diabetes and cardiovascular diseases in their later life in Chinese and other populations [1–4]. Offspring born to GDM-complicated pregnancy were more prone to develop childhood obesity, impaired glucose tolerance and increased cardiovascular risk profile during adolescence and early adulthood [5]. The prevalence of GDM has been increasing globally including in China. In Tianjin, a cosmopolitan city near Beijing in North China, the prevalence of GDM has increased from 2.3% in 1999 to 6.8% in 2008 [6,7]. Despite the rising prevalence, comparison of prevalence data across regions/areas is hampered by differences in screening procedures and diagnostic criteria for GDM and lack of quality population-based studies. In this light, the International Association of Diabetes and Pregnancy Study Group (IADPSG) [8] reviewed the evidence of the landmark study of the Hyperglycaemia and Adverse Pregnancy Outcomes (HAPO) Survey [1] and recommended use of a new set of diagnostic criteria for GDM by one-step approach performing a 75-gram 2-hour oral glucose tolerance (OGTT).

In the past two decades, China has witnessed rapid lifestyle and socioeconomic changes with increasing westernization, characterized by changes in dietary intake and physical inactivity [9]. These factors have been implicated in the rapidly increasing prevalence of diabetes in general population in China, reported to be 11.6% in a national survey in 2010 [10]. Whether these secular changes in lifestyle patterns and associated risk factors might also be relevant to the increasing prevalence of GDM has not been systematically studied.

In 1998, our group launched the Tianjin Study of Diabetes in Pregnancy [6]. The study established a universal GDM screening and management system within the shared antenatal care system in Tianjin, China. The screening system included administration of a 50-gram 1-hour glucose challenge test (GCT) to all pregnant women at primary care hospitals. Women with 1-hour plasma glucose (PG) ≥7.8 mmol/L were referred to Tianjin Women and Children’s Health Center to undergo a standard 75-gram 2-hour OGTT for diagnosis of GDM using the World Health Organization (WHO) criteria [11]. Soon after publication of the recommendation of IADPSG in 2010, the antenatal care system adopted the IADPSG criteria to interpret the OGTT results. To minimize changes in logistics, the two-step screening procedure and 3-tier management system were maintained which do not comply with the IADPSG one-step criteria.

Based on this well-established universal GDM screening and management system, we estimated the latest prevalence of GDM using both the WHO and the IADPSG criteria and associated risk factors with comparison to data collected 12 years ago in a similar setting.

## Methods

### Study Population and Setting

Tianjin is the fourth largest city with over 13 million residents, located in Northern China, 130 kilometres southeast of Beijing and is directly under the administration of the central government of China. Tianjin has six central urban districts, one new urban district, four suburban districts and five counties. There are about 4.3 million residents living in the 6 central urban districts, where antenatal care is delivered through a 3-tier prenatal care system consisting of 65 primary care hospitals (the 1^st^ tier), 6 district-level women and children’s health centers (WCHCs) and other secondary obstetric hospitals (the 2^nd^ tier), and a city-level women and children’s health center, i.e., Tianjin Women and Children’s Health Center and other tertiary obstetric hospitals (the 3^rd^ tier). Tianjin Women and Children’s Health Center was the coordinating institution of the 3-tier prenatal care system. The 65 primary care hospitals governed by 6 district WCHCs were the antenatal care providers for pregnant women from registration up to the 32^nd^ gestational week. After that, these pregnant women were referred to one of the 25 secondary or tertiary hospitals at their choice for continued care till delivery.

In 2009, Tianjin set up a computerized Maternal and Child Health Information System, consisting of records of antenatal examinations, delivery data and regular health examinations of children up to 6 years of age with the central database managed by Tianjin Women and Children’s Health Center. At registration for pregnancy, all pregnant women received a unique identification number that links their antenatal care information recorded by different antenatal care providers.

From October 2010 to August 2012, we set up a cohort of pregnant women and their infants. During this period, 19669 pregnant women within 12 gestational weeks were registered. After excluding 1080 women who did not have GCT at 24–28 weeks of gestation, 18589 women were included in the analysis to estimate the prevalence of GDM with a response rate of 94.5%. Of these, 781 women had a positive GCT but absented from the standard OGTT. These women were included in the estimation of prevalence of GDM but excluded in the analysis to identify risk factors for GDM. Ethics approval was obtained from the Ethics Committee for Clinical Research of Tianjin Women and Children’s Health Center and written informed consent was obtained from all pregnant women.

### Screening for and diagnosis of GDM

A 50-gram 1-hour GCT was used to screen pregnant women for GDM between 24 and 28 weeks of gestation at primary care hospitals. Non-fasting venous blood was taken 60 minutes after the ingestion of 200 ml of 25% glucose solution to measure PG. Women with PG ≥7.8 mmol/L were referred to Tianjin Women and Children’s Health Center for a standard 75-gram 2-hour OGTT. After overnight fasting of at least 8 hours, the women ingested 300 ml of 25% glucose solution in the morning, and venous blood was drawn at fasting, 1 hour and 2 hours after the glucose load. All blood samples were measured at the laboratory of Tianjin Women and Children’s Health Center using an automatic analyzer (Toshiba TBA-120FR, Japan) with a coefficient of variance <2.59%. The IADPSG criteria were used for diagnosis of GDM defined by any one of the cutoff values: fasting plasma glucose (FPG) ≥5.1 mmol/L and/or 1-hour PG ≥10.0 mmol/L and/or 2-hour PG ≥8.5 mmol/L [8]. For comparison with the prevalence of GDM in this population in 1999 [6], we also estimated the prevalence using the 1999 WHO criteria [11]. The latter defined diabetes as FPG≥7.0 mmol/L and/or 2 h PG≥11.1 mmol/L, impaired glucose tolerance (IGT) as FPG<7.0mmol/L and 2-h PG≥7.8 mmol/L but <11.1 mmol/L and impaired fasting glucose (IFG) as FPG≥6.1 mmol/L but <7.0 mmol/L and 2-h PG<7.8mmol/L. Using the 1999 WHO criteria, GDM was defined as having either diabetes, IGT or IFG.

### Anthropometric and clinical measurements

Before the study, training workshops were held to standardize the anthropometric and clinical measurements. Body height and weight were measured with a beam balance scale (RGZ-120, Jiangsu Suhong Medical Instruments Co., China) following a standardized procedure. Sitting blood pressure (BP) was measured using a calibrated mercury sphygmomanometer (XJ11D, Shanghai Medical Instruments Co., China) after at least 10 min of rest. Body weight at the registration was treated as pre-pregnancy weight due to small weight gain during the first 12 gestational weeks [12]. Weight gain from pre-pregnancy to GCT was estimated as the difference in body weight from registration to GCT. Body mass index (BMI) was calculated as weight in kilogram divided by the square of body height in meter. Obesity and overweight were defined by the criteria recommended by the Working Group on Obesity in China (WGOC) [13], i.e., underweight: BMI <18.5 kg/m^2^, normal weight: BMI 18.5 to 23.9 kg/m^2^, overweight: BMI 24.0 to 27.9 kg/m^2^ and obesity: BMI ≥28.0 kg/m^2^.

### Data Collection

Data were collected longitudinally by care providers or self-administered questionnaires and/or retrieved from the database of Maternal and Child Health Information System. At registration for pregnancy at primary care hospitals, the obstetrician and the woman separately completed a questionnaire to collect demographic and socioeconomic information, lifestyle, personal and family history of disease, obstetric history and this pregnancy-related information, including maternal birth date, date of the last menstrual period, date of registration, diabetes in first degree relatives, parity, education attainment, nationality, habitual use of tobacco and use of alcohol before pregnancy. At the screening visit for GDM, both care providers and women were asked to fill in another questionnaire for record of results of GCT, physical examinations, pregnancy-related medical information and lifestyle changes during this pregnancy.

Maternal age at registration was stratified into <30 years, 30 to <35 years and ≥35 years. Habitual smoking before pregnancy or during pregnancy was defined as continuously smoking one or more cigarettes per day for at least 6 months before pregnancy or smoking one or more cigarettes per day during pregnancy.

### Statistical Analyses

The method to estimate the prevalence of GDM in 1999 study was used to estimate the prevalence of GDM in the current study [6], i.e., the adjusted prevalence of GDM was calculated for the whole cohort of women by including an estimate of the proportion of women with a positive GCT who did not have OGTT but would have been expected to have GDM. The proportion of GDM for women with a positive GCT who did not have OGTT was estimated using the proportion of women with positive GCT who were diagnosed with GDM for each level of a positive GCT [6]. Continuous variables were compared using Student t-test if their normal distribution was not rejected. Categorical variables were compared using Chi-square test or Fisher’s exact test where appropriate. Binary logistic regression was performed to obtain odds ratios (ORs) and 95% confidence intervals (CIs) of risk factors for GDM in univariable and multivariable analysis. We used Pearson correlation analysis to verify that there were no highly correlated covariates in the multivariable model (correlation coefficient <0.60) [14]. P values and 95%CIs of ORs were adjusted for multiple comparisons using Ryan-Holm step-down Bonferroni procedure [15,16]. Comparisons of GDM risk factors between the pregnant women enrolled in 1999 study [6] and the women in this study were performed. All statistical analyses were conducted using IBM SPSS Statistics 20.0 (IBM SPSS, Chicago, IL). P values <0.05 for a two-tailed test were considered to be statistically significant.

## Results

### Prevalence of GDM defined by the IADPSG and the WHO criteria

The crude prevalence of GDM by the 1999 WHO criteria was 6.8%. A shift from the 1999 WHO to the IADPSG criteria increased the crude prevalence to 7.7% (Table 1). The adjusted prevalence of GDM was calculated based on the same proportions of GCT levels of women who had a positive GCT and further underwent the OGTT. The adjusted prevalence was 8.1% (7.4% with IGT, 0.6% with diabetes and 0.1% with IFG) based on the 1999 WHO criteria. The use of the IADPSG criteria increased the adjusted prevalence to 9.3%.

**Table 1:** Prevalence of gestational diabetes diagnosed by the 1999 WHO criteria and the IADPSG criteria.

	The 1999 WHO criteria defined GDM	The IADPSG criteria defined GDM
Cases with GCT ≥7.8 mmol/L and with OGTT, n (%)		
≥7.8–<9.1, mmol/L	619(32.7%)	716(37.8%)
≥9.1–<10.1, mmol/L	291(47.4%)	329(53.7%)
≥10.1–<11.1, mmol/L	142(55.9%)	161(63.4%)
≥11.1–<12.1, mmol/L	65(66.3%)	75(76.5%)
≥12.1 mmol/L	76(80.8%)	78(83.0%)
Cases with GCT<7.8 mmol/L(n)	13*	19*
Total GDM case identified by OGTT(n)	1206	1378
Crude prevalence (%)	6.8%†	7.7%†
Expected cases with GCT but without OGTT, n (%)		
≥7.8–<9.1, mmol/L	192(32.7%)	222(37.8%)
≥9.1–<10.1, mmol/L	47(47.4%)	53(53.7%)
≥10.1–<11.1, mmol/L	30(55.9%)	34(63.4%)
≥11.1–<12.1, mmol/L	18(66.3%)	21(76.5%)
≥12.1 mmol/L	13(80.8%)	13(83.0%)
Expected cases with GCT but without OGTT (n)	300	343
Total GDM cases identified by OGTT and expected in women with a positive GCT but without OGTT(n)	1506	1721
Adjusted prevalence (%)	8.1%††	9.3%††

Abbreviations: WHO, World Health Organization; IADPSG, International Association of Diabetes and Pregnancy Study Group; GCT, glucose challenge test; OGTT, oral glucose tolerance test.

* 40 women with GCT<7.8 mmol/L were referred to have OGTT because they had GCT before the 19th gestational week and meanwhile had a family history of diabetes.

† The total sample size of 17808 women was used as the denominator in calculation of the prevalence.

†† The total sample size of 18589 women was used as the denominator in calculation of the prevalence.

### Characteristics of the study population

Amongst the 17808 women who underwent GCT with OGTT if needed, the mean gestational age at registration was 9.8 (SD: 1.6) weeks, mean age at registration was 28.5 (SD: 2.8) years, mean height was 163.2 (SD: 4.7) cm, mean pre-pregnancy BMI was 22.3 (SD: 3.4) kg/m^2^, and mean diastolic/systolic BP at registration were 68.4/105.6 (SD: 7.7/10.6) mmHg. Of all women, 19.5% were overweight and 6.3% were obese. The GCT was performed at a mean gestational week of 24.8 (SD: 2.4) and the mean GCT 1-hour PG values was 6.6 (SD: 1.5) mmol/L. Women with GDM were older, had higher pre-pregnancy BMI, shorter body height and higher systolic/diastolic BP, were more likely to have Han-nationality, parity≥1, multiple pregnancies, a family history of diabetes and habitual use of tobacco than women without GDM (Table 2).

**Table 2:** Clinical and biochemical characteristics of subjects stratified by gestational diabetes mellitus diagnosed by the IADPSG criteria.

	Non GDM	GDM	P-value
N	16430	1378	
Variables at registration for pregnancy			
Age, year	28.4±2.8	29.5±3.2	<0.001*
Age group, year			<0.001**
<30	12570(76.5%)	899(65.2%)	
≥30–<35	3460(21.1%)	398(28.9%)	
≥35	400(2.4%)	81(5.9%)	
Body height, cm	163.2±4.7	162.7±4.7	<0.001*
Body height group, cm			<0.001**
<160	2659(16.2%)	275(20.0%)	
≥160–<165	7202(43.8%)	603(43.8%)	
≥165	6565(40.0%)	500(36.3%)	
Pre-pregnancy BMI, kg/m^2^	22.1±3.3	24.1±3.9	<0.001*
Pre-pregnancy BMI group, kg/m^2^			<0.001**
<18.5	1716(10.4%)	48(3.5%)	
≥18.5–<24	10748(65.4%)	697(50.6%)	
≥24–<28	3027(18.4%)	442(32.1%)	
≥28	933(5.7%)	191(13.9%)	
Gestational age at registration, weeks	9.8±1.6	9.8±1.6	0.820*
Diastolic BP, mmHg	68.3±7.7	70.5±8.0	<0.001*
Systolic BP, mmHg	105.4±10.6	108.4±11.0	<0.001*
Parity≥1	515(3.1%)	59(4.3%)	0.021**
Han-nationality	15676(95.4%)	1334(96.8%)	0.016**
Single pregnancy	16094(99.0%)	1349(98.5%)	0.047**
Family history of diabetes in first degree relatives	1232(7.5%)	191(13.9%)	<0.001**
Education attainment			0.258**
High school and below	2590(15.8%)	237(17.2%)	
Junior college	4597(28.0%)	363(26.4%)	
University	7885(48.1%)	673(49.0%)	
More than University	1333(8.1%)	101(7.4%)	
Variables during pregnancy			
Gestational age at GCT, weeks	24.8±2.5	24.8±1.8	0.127*
Weight gain from pre-pregnancy to GCT, kg	7.5±3.4	7.5±3.6	0.467*
Smoking habit			
Habitual smoker before pregnancy†	483(2.9%)	57(4.1%)	0.013**
Habitual Smoker during pregnancy††	99(0.6%)	11(0.8%)	0.373**
Alcohol drinking habit			
Alcohol drinker before pregnancy	5048(30.7%)	425(30.8%)	0.928**
Alcohol drinker during pregnancy	119(0.7%)	10(0.7%)	0.995**

Abbreviations: IADPSG, International Association of Diabetes and Pregnancy Study Group; BMI, body mass index; BP, blood pressure; GCT, glucose challenge test.

Data are reported in mean ± SD or number (%).

* Derived from Student’s t- test

** Derived from Chi-square test or Fisher’s exact test.

† Defined as having continuously smoked one or more cigarettes per day for at least six months before pregnancy.

†† Defined as having smoked one or more cigarettes per day during pregnancy.

### Risk factors for GDM

On univariable analysis, older age, pre-pregnancy overweight/obesity, Han-nationality, higher systolic BP, family history of diabetes, parity≥1, multiple pregnancies and habitual smoking before or during pregnancy were positively associated with the risk of GDM (Table 3). After adjustment for covariables, older age, pre-pregnancy overweight/obesity, Han-nationality, higher systolic BP and family history of diabetes remained significantly associated with the risk of GDM. After adjustment, habitual smoking before or during pregnancy became marginally significant, parity≥1 and multiple pregnancies were no longer significant but weight gain from pre-pregnancy to GCT was significantly associated with the risk of GDM (Table 4).

**Table 3:** Univariable odds ratios of potential risk factors for gestational diabetes mellitus diagnosed by the IADPSG criteria.

	N (%)	Odds ratio	95%CI	P-value
Age at registration, year		1.13	1.11–1.15	<0.001
Pre-pregnancy BMI, kg/m^2^		1.16	1.14–1.18	<0.001
Han nationality	1334(7.8%)	1.46	1.07–1.99	0.017
Parity≥1	59(10.3%)	1.38	1.05–1.82	0.021
Systolic BP at registration, per 10 mmHg		1.31	1.24–1.38	<0.001
Family history of diabetes in first degree relatives	191(13.4%)	2.00	1.69–2.34	<0.001
Education attainment				
High school and below	237(8.4%)	Reference		
Junior college	363(7.3%)	0.86	0.70–1.06*	0.270*
University	673(7.9%)	0.93	0.80–1.09*	0.377*
More than University	101(7.0%)	0.83	0.63–1.09*	0.254*
Non-single pregnancy	21(11.7%)	1.59	1.00–2.51	0.049
Weight gain from pre-pregnancy to GCT, per kg		0.99	0.98–1.01	0.467
Habitual smoker before or during pregnancy	60(10.5%)	1.42	1.08–1.86	0.012
Alcohol drinker before or during pregnancy	425(7.8%)	1.01	0.89–1.13	0.928

N (%), number of cases (% of number at risk).

Abbreviations: IADPSG, International Association of Diabetes and Pregnancy Study Group; BMI, body mass index; BP, blood pressure; GCT, glucose challenge test.

* P values and 95%CIs of ORs were adjusted for multiple comparisons by Ryan-Holm step-down Bonferroni procedure.

**Table 4:** Multivariable odds ratios of potential risk factors for gestational diabetes mellitus diagnosed by the IADPSG criteria.

	N (%)	Odds ratio	95%CI	P-value
Age at registration, year		1.12	1.10–1.14	<0.001
Pre-pregnancy BMI, kg/m^2^		1.15	1.13–1.17	<0.001
Han nationality	1334(7.8%)	1.54	1.12–2.11	0.008
Parity≥1	59(10.3%)	0.84	0.62–1.12	0.234
Systolic BP at registration, per 10 mmHg		1.11	1.05–1.17	<0.001
Family history of diabetes in first degree relatives	191(13.4%)	1.61	1.36–1.91	<0.001
Education attainment				
High school and below	237(8.4%)	Reference		
Junior college	363(7.3%)	0.96	0.79–1.18*	1.000*
University	673(7.9%)	1.03	0.87–1.27*	0.731*
More than University	101(7.0%)	0.86	0.63–1.18*	0.752*
Non-single pregnancy	21(11.7%)	1.13	0.70–1.82	0.631
Weight gain from pre-pregnancy to GCT, per kg		1.02	1.01–1.04	0.005
Habitual smoker before or during pregnancy	60(10.5%)	1.30	0.97–1.74	0.080
Alcohol drinker before or during pregnancy	425(7.8%)	0.96	0.84–1.08	0.479

Variables adjusted in the multivariable analysis included the variables listed in the model.

Abbreviations: IADPSG, International Association of Diabetes and Pregnancy Study Group; BMI, body mass index; BP, blood pressure; GCT, glucose challenge test.

* P values and 95%CIs of ORs were adjusted for multiple comparisons by Ryan-Holm step-down Bonferroni procedure.

### Comparison of prevalence of risk factors between present study and 1999 survey

Compared to our previous survey (n = 9474) in 1999 [6], the women (n = 18589) in the present study were older, had a higher pre-pregnancy BMI, higher systolic/diastolic BP at registration for pregnancy and less weight gain from pre-pregnancy to GCT per week. They were also more likely to be multiparous and habitual smokers during pregnancy. During a span of 12 years, the prevalence of pre-pregnancy overweight/obesity had markedly increased from 14.8% in 1999 to 26.4% in 2010–2012. The proportions of women with age≥30 years at pregnancy also increased from 9.9% to 22.8% (Table 5).

**Table 5:** Increased prevalence of risk factors among pregnant women in Tianjin from 1999 to 2010–2012.

	The 1999 study	This study	P-value
N	9474	18589	
Age at pregnancy, year†	26.3±3.2	28.3±2.9	<0.001*
Age≥30 years at pregnancy†	933(9.9%)	4239(22.8%)	<0.001**
Pre-pregnancy BMI, kg/m^2^	21.2±2.9	22.3±3.4	<0.001*
Pre-pregnancy BMI≥24 kg/m^2^	1400(14.8%)	4901(26.4%)	<0.001**
Systolic BP at registration, mmHg	103.3±10.5	105.6±10.6	<0.001*
Diastolic BP at registration, mmHg	67.4±7.4	68.5±7.7	<0.001*
Parity ≥1	231(2.4%)	606(3.3%)	<0.001**
Han-nationality	9085(95.9%)	17763(95.6%)	0.189**
Family history of diabetes in first degree relatives	789(8.3%)	1527(8.2%)	0.744**
Weight gain before GCT, kg per week††	0.36±0.15	0.30±0.14	<0.001*
Habitual smoker during pregnancy‡	17(0.2%)	113(0.6%)	<0.001**

Data are reported in mean ± SD or number (%).

* Derived from Student’s t- test;

** Derived from Chi-square test or Fisher’s exact test.

Abbreviations: BMI, body mass index; BP, blood pressure; GCT, glucose challenge test.

† To make age between the 1999 study and the current study comparable, age was re-calculated as at pregnancy.

†† Due to difference in gestational age at GCT between the two studies, adjustment for gestational age at GCT was made for the comparison.

‡ Only habitual smoking during pregnancy was available in both the study in 1999 and the current study and therefore compared between the two periods.

## Discussion

In this population-based study in Tianjin, China, the prevalence of GDM in 2010–2012 was 8.1% using the 1999 WHO criteria which further increased to 9.3% if the IADPSG criteria were used.

Using the 1999 WHO criteria, we reported a 2.3% of prevalence of GDM in 1999 [6]. In 2008, we witnessed a rapid increase in the prevalence of GDM in the same population to 6.8% [7]. Using the same criteria, the prevalence had increased to 8.1% in this 2010–2012 survey showing the continuing and rising trend of GDM in this population. While in both surveys conducted in 1999 and 2010–2012, the risk factors for GDM remained largely similar, and there were marked increases in the prevalence of these risk factors, notably, obesity/overweight, advanced maternal age and increased BP. These secular changes in traditional risk factors might at least partially account for the increased prevalence of GDM in Tianjin. A shift of the diagnostic criteria from the 1999 WHO to the IADPSG criteria further resulted in a rise in the prevalence of GDM.

In China, two studies had reported prevalence of the IADPSG criteria-defined GDM of 14.7% in 2004–2009 (n = 14593) and 10.9% (n = 6201) in 2008–2011[17,18]. However, both studies were conducted in tertiary care setting which were less representative of the general pregnant women population with potential bias such as higher medical risk or higher socioeconomic status.

Consistent with our previous study and other studies [6,19,20], advanced maternal age was a strong risk factor for GDM. Compared to the 1999 survey, there was a 2-year increase in age at the time of pregnancy in the present study with the proportion of women in ≥30 year age group being increased from 9.9% to 22.8%. Obesity/overweight was a major risk factor for type 2 diabetes and GDM [21,22]. In this analysis, the pre-pregnancy BMI had increased by 1.1 kg/m^2^ during this 12-year period with proportion of overweight/obesity being increased from 14.8% to 26.4%. Probably due to shared aetiologies or other yet to be identified mediators, high BP was shown to independently predict type 2 diabetes [23]. Here, BP either as a continuous variable within a normal range or a category (pre-hypertension and hypertension) was also a risk factor for GDM [24,25]. Importantly, there was a large secular increase of 2.3 mmHg in systolic BP over a 12-year period in pregnant women in Tianjin.

In concordance with previous reports [26,27], weight gain from pre-pregnancy to GCT also predicted GDM in our present analysis. In the 1999 survey, cigarette smoking during pregnancy was a risk factor for GDM [6] while in the current study, habitual smoking before or during pregnancy increased the risk of GDM. Although only 0.6% of these women were habitual smokers during pregnancy, there was a 3-fold increase compared to 0.2% in the 1999 survey. Consistent with our previous study [6] and other studies [28,29], family history of diabetes was highly associated with GDM. However, the odds ratio of GDM for family history of diabetes in first degree relatives had decreased from 3.46(2.43–4.93) to 1.61(1.36–1.91), probably due to increased prevalence of other risk factors. Although high parity was a well recognized risk factor for GDM [29,30]. However, no association was found in our study, possibly in part due to the one-child policy in China with few women being multiparous.

The rapid urbanization in China during the past three decades had brought about secular changes in lifestyles resulting in a rapid increase in the prevalence of obesity and metabolic syndrome [31,32]. While these risk factors, albeit highly modifiable, had contributed largely to the rising prevalence of diabetes in China [10], the same factors along with other risk factors such as advanced maternal age, overweight /obesity, increased BP and smoking had also led to the rising prevalence of GDM in our population in the past 12 years. Given to the high risk for young-onset diabetes in women with GDM and the potential effects of GDM on cardiometabolic risks in offspring born to mothers with GDM, the public, family and personal implications of this rising prevalence of GDM is particularly concerning. To prevent and control this public health emergency, large scale awareness, surveillance and prevention programs focusing on lifestyle modification before pregnancy and during early pregnancy in young women, notably those with risk factors, such as obesity and positive family history, need to be introduced to reduce the burden of GDM in China and to break the vicious cycle of ‘diabetes begetting diabetes’.

This study has several strengths and limitations. The strength of the study is its nature of a real-world population-based study with detailed profile collected using similar methods in 1999 for comparison. The major limitation is that the IADPSG recommended identifying GDM using a one-step OGTT approach [8] whilst our 3-tier’s antenatal care system has developed and used a two-step procedure to detect GDM. Logistic problems prevented us to completely shift from the two-step procedure. Due to this pre-screening procedure, we might have underestimated the true prevalence of GDM in our population with those women with fasting hyperglycaemia alone missed. Nevertheless, the distributions of clinical and biochemical characteristics were similar in 781 women who had a positive GCT but absented from the OGTT and 2953 women who had a positive GCT and further underwent the OGTT (data were not shown), which suggests that major bias for the adjusted prevalence of GDM due to the non-respondents was unlikely. Moreover, use of this adjustment approach that was used in the 1999 survey [6] also makes it possible to compare the prevalence in Tianjin over time.

In conclusion, in this population-based study, the prevalence of GDM in Tianjin, China had increased substantially from 2.3% in 1999 to 8.1% in 2010–2012 using the WHO criteria, which increased to 9.3% if the IADPSG criteria used. In parallel, we observed a huge increase in pre-pregnancy BMI, obesity/overweight and BP at registration for pregnancy, and increased age at pregnancy in pregnant women over the 12-year period. While prevention of obesity/overweight among young women should be a major public health priority in China to control the rising trend of GDM, randomized controlled trials on screening strategy for GDM and its intervention will be needed to inform clinical practice.
